# Constructing Abundant Oxygen-Containing Functional Groups in Hard Carbon Derived from Anthracite for High-Performance Sodium-Ion Batteries

**DOI:** 10.3390/nano13233002

**Published:** 2023-11-22

**Authors:** Yaya Xu, Donglei Guo, Yuan Luo, Jiaqi Xu, Kailong Guo, Wei Wang, Guilong Liu, Naiteng Wu, Xianming Liu, Aimiao Qin

**Affiliations:** 1 Key Laboratory of New Processing Technology for Nonferrous Metal & Materials, Ministry of Education, Guilin University of Technology, Guilin 541004, China; xuyayachonga@163.com (Y.X.); luo1500935739@163.com (Y.L.); 15397973347@163.com (K.G.); 2 Key Laboratory of Function-Oriented Porous Materials, College of Chemistry and Chemical Engineering, Luoyang Normal University, Luoyang 471934, China; 18836032666@163.com (J.X.); glliu@tju.edu.cn (G.L.); wunaiteng@gmail.com (N.W.); 3 Ningbo Key Laboratory of Agricultural Germplasm Resources Mining and Environmental Regulation, College of Science and Technology, Ningbo University, Cixi 315300, China; wangwei4@nbu.edu.cn; 4 Guangxi Key Laboratory of Optical and Electronic Materials and Devices, Guilin University of Technology, Guilin 541004, China

**Keywords:** sodium-ion batteries, hard carbon, anthracite, pre-oxidation

## Abstract

Hard carbon is regarded as one of the greatest potential anode materials for sodium-ion batteries (SIBs) because of its affordable price and large layer spacing. However, its poor initial coulombic efficiency (ICE) and low specific capacity severely restrict its practical commercialization in SIBs. In this work, we successfully constructed abundant oxygen-containing functional groups in hard carbon by using pre-oxidation anthracite as the precursor combined with controlling the carbonization temperature. The oxygen-containing functional groups in hard carbon can increase the reversible Na^+^ adsorption in the slope region, and the closed micropores can be conducive to Na^+^ storage in the low-voltage platform region. As a result, the optimal sample exhibits a high initial reversible sodium storage capacity of 304 mAh g^−1^ at 0.03 A g^−1^, with an ICE of 67.29% and high capacitance retention of 95.17% after 100 cycles. This synergistic strategy can provide ideas for the design of high-performance SIB anode materials with the intent to regulate the oxygen content in the precursor.

## 1. Introduction

Rechargeable sodium-ion batteries (SIBs) have been thought to be a potential candidate for lithium-ion batteries (LIBs) due to their affordable cost and rich reserves of sodium [1,2,3]. To some extent, SIBs show superior advantages in low-speed electric vehicles and grid electricity storage systems compared to LIBs. At present, successful research on the electrode materials of LIBs has enabled the development of SIBs due to the similar properties of Na and Li and their similar operational mechanisms [4,5,6]. Unfortunately, graphite, the anode material used for commercial LIBs, is unsuitable for sodium storage due to: (a) the radius of Na^+^ being wider than the radius of Li^+^ (1.02 Å vs. 0.76 Å); (b) the intense regional interaction between Na^+^ and graphene layers; and (c) the narrow interlayer spacing of graphite, which limits the storage capacities and diffusion kinetics of Na^+^ to some extent [7]. Hence, it is still very challenging to develop high-performance and suitable anode materials at low cost for SIBs.

Thus far, numerous types of anode materials have been explored for SIBs, such as carbon materials [8], metal [9,10], and mixed metal oxides [11,12]. Carbon materials have been extensively researched since the early 1980s owing to their inexpensiveness, high abundance, and outstanding electrochemical characteristics as suitable anode electrode materials for power sources of rechargeable batteries or supercapacitors. So far, numerous carbon materials with different structures have been explored, such as soft carbons [13], hard carbons [14], and hybrid carbons [15]. Among them, hard carbon materials have a more competitive cost, with large interlayer spacing, an irregular structure, and low operation potential. They have been recognized as the preferred anode material for SIBs [16]. At present, the use of precursors in large amounts and at a low cost is one of the common methods to produce hard carbon materials, such as coal [17,18,19], resin [20,21,22], asphalt [23], and oxygen-rich biomass [24,25,26]. As a natural mineral with the greatest amount of carbon, coal is rich in resources and is mainly used in power, chemical, and other energy-intensive industries. Anthracite, as a type of coal-based material, has the characteristics of a clear and orderly graphite structure with high carbon content compared with other coal resources [27]. Through the simple one-step pyrolysis of anthracite, Liu et al. prepared carbon materials with a mixed structure of order and disorder, which reached 384.5 mAh g^−1^ at a current density of 100 mA g^−1^ when used as an LIB anode [28]. Hu et al. carried out a simple carbonization process on anthracite to prepare an SIB anode, which provided a high sodium storage capacity of 222 mAh g^−1^ at 30 mA g^−1^ with good magnification performance and a long cycle life [29]. During the process of carbonization, the structure of functional groups changes, and the oxygen groups not only play a significant role in regulating the structure of the carbon anode, but also participate in the adsorption of Na^+^ [30,31]. Xie et al. found that when the precursor being utilized contains unsaturated oxygen functional groups (OFGs), the bonds (-C=O) easily break and volatilize during carbonization, thus forming a more ordered structure [32]. Depending on the mechanism of the redox reaction (C=O + Na^+^ + e^−^↔C-O-C-Na) between OFGs and alkali metal ions in the carbonaceous electrode, the introduced OFGs can provide additional active sites for Na^+^ storage [33]. Luo reported a method of two-step “thermal-exfoliation” to retain more C=O groups by adjusting the number of OFGs on the surfaces of graphene nanosheets, thus generating enough active sites, and the prepared sodium electric anode provided a 603 mAh g^−1^ reversible capacity at a current density of 0.05 A g^−1^ [17]. Shao et al. prepared nano-cellular carbon foam (NCCF) with oxygen functional groups on the surface by using concentrated H_2_SO_4_/HNO_3_ mixed acid, which provided a capacity of 152 mAh g^−1^ at a current density of 0.1 A g^−1^, and the capacity retention was 90% after more than 1600 cycles [34]. By adjusting the content of OFGs in the precursor, the material morphology can effectively improve the electrochemical performance, but if this is applied in practice, it is necessary to reduce the cost and flow of the process.

In this work, we propose an effective strategy to prepare low-cost hard carbon (HC) anode materials by using pre-oxidation anthracite as a precursor. The carboxyl groups (C=O and -COOH) were successfully introduced into the hard carbon material, and the content of carboxyl groups (C=O) increased. The optimal hard carbon was achieved by combining the pre-oxidation and carbonization temperature, and it exhibited excellent electrochemical performance (304 mAh g^−1^ at 0.03 A g^−1^ and high capacitance retention of 95.17% after 100 cycles). The outstanding electrochemical performance may be attributed to the abundant oxygen-containing functional groups (-COOH or C=O), which can produce more adsorption sites.

## 2. Materials and Methods

### 2.1. Material Synthesis

The anthracite used in this work was pretreated. Firstly, the crushed anthracite was washed several times with deionized water (50 °C), and then impregnated and stirred in 5 mol L^−1^ potassium hydroxide (KOH) with constant stirring for 12 h. Secondly, the anthracite was dispersed in 5 mol L^−1^ hydrochloric acid (HCL) for 24 h and washed with deionized water until the PH was 7. Finally, the anthracite was dried in an oven at 80 °C for 24 h for further use, and the impurity was removed accordingly (Figure S1). The pretreated anthracite was pre-oxidated in a muffle furnace at 300 °C for 3 h under an air atmosphere. Then, the obtained precursor was annealed at 900, 1100, and 1300 °C for 3 h with a heating rate of 2 °C min^−1^ under an Ar atmosphere (denoted as A-HC900, A-HC1100, and A-HC1300), respectively. For comparison, bulk hard carbon was synthesized using pristine anthracite without pre-oxidation as a precursor, then annealed at 1100 °C for 3 h (denoted as B-HC1100).

### 2.2. Material Characterization

The morphology of the samples was studied by scanning electron microscopy (SEM, S4800) and transmission electron microscopy (JEM-2100F). The crystalline structure of the hard carbon material was determined by X-ray diffraction (XRD) (Cu Kα radiation λ = 1.54 Å, 2θ = 10–60°), as well as Raman spectroscopy, using a 532 nm excitation wavelength. Fourier transform infrared (FTIR) spectra were used with a Nicolet6700 (Thermos Fisher Scientific, Waltham, MA, USA) to analyze the functional groups of the samples. Nitrogen adsorption–desorption at 77 K was tested on ASAP 2020 to characterize the micropores and mesopores of the hard carbon. The Brunauer–Emmette–Teller (BET) theory was used to calculate the specific surface area and pore size distribution. The elements of the carbon materials were analyzed by X-ray photoelectron spectroscopy with Al Kα radiation.

### 2.3. Electrochemical Measurements

The working electrode was prepared by mixing the hard carbon with super P (specialty carbon black) and polyvinylidene fluoride (PVDF) binder at a weight ratio of 7:2:1 in N-methyl-2- pyrrolidone (NMP), then coated on Cu foil followed by drying in a vacuum at 120 °C for 12 h. Then, the sample was cut into slices with diameters of 12 mm using a manual slicer. The electrolyte was 1.0 mol L^−1^ NaClO_4_ in ethylene carbonate (EC) and dimethyl carbonate (DEC), with 1:1 and 5 vol% fluoroethylene carbonate volume ratios. The glass fiber membrane (GF/A, Whatman, Maidstone, UK) was used as the separator, and the sodium metal was used as the counter electrode. The electrochemical properties of the HC were tested using 2032-type coin cells, which were assembled in an argon-filled glove box. The coin cells were tested using a Neware GCD testing system at different current densities to obtain galvanostatic charging and discharging profiles. The CV measurement was carried out in a voltage range of 0.01–3 V (versus Na/Na^+^), and the EIS was measured in a frequency range of 0.01–100,000 Hz.

## 3. Results

### 3.1. Synthesis and Characterization of HCs

**Figure 1:**
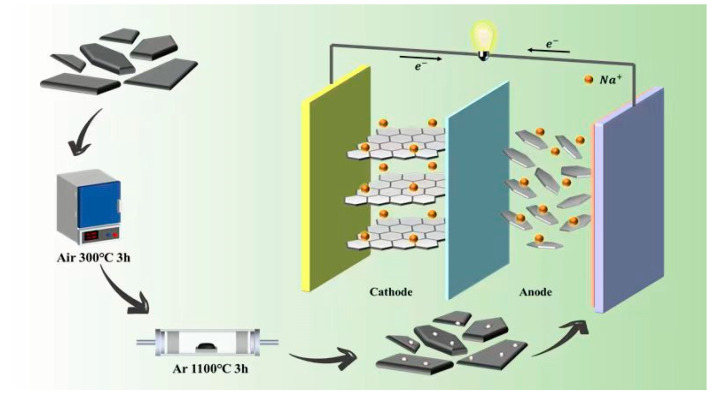
Schematic illustration of the fabrication of A-HC1100 for SIBs.

**Figure 2:**
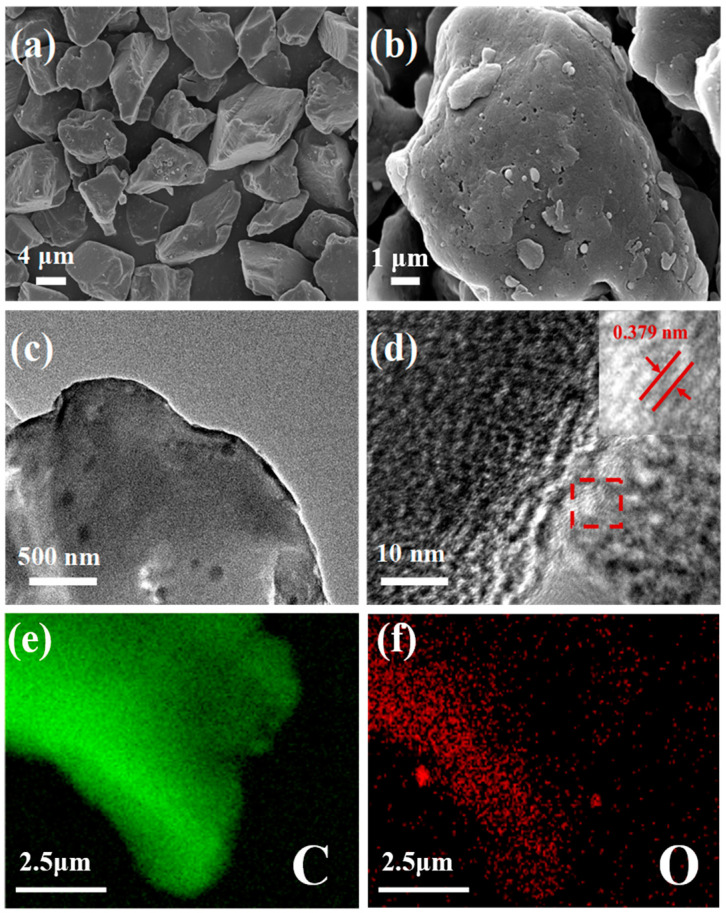
(**a**,**b**) FESEM images; (**c**,**d**) HRTEM images; and (**e**,**f**) elemental mapping images of A-HC1100.

A schematic illustration of the fabrication of A-HC1100 is shown in Figure 1. First, the anthracite was pre-oxidated in a muffle furnace at 300 °C for 3 h under an air atmosphere, and then annealed at 1100 °C for 3 h under an Ar atmosphere with a heating rate of 2 °C min^−1^ (denoted as A-HC1100). The morphological characteristics of the as-prepared HC were observed by FESEM and TEM. It was observed that A-HC1100 had an irregular block structure (Figure 2a,b). When the carbonization temperature changed, uniform small balls gradually overflowed on the surface of the material (Figure S2a–c), indicating that the carbonization temperature had a certain influence on the morphology of the HC. As we all know, less crystalline materials with finer particle sizes at lower synthesis temperatures are suitable for battery electrodes due to the short diffusion paths [35]. However, the synthesis temperature does not have a significant influence on HC in terms of particle size. The TEM images of A-HC1100, B-HC1100, A-HC900, and A-HC1300 are compared in Figure 2c and Figure S3a–c. As can be seen, all samples had a short-range ordered carbon layer structure, different from long-range ordered graphite. However, B-HC1100 had a smooth surface and no defects, while the samples derived from pre-oxidized anthracite showed deeper deformation of the graphite layer along with arresting defects, which may be attributed to the introduction of oxygen atoms distorting the graphite sheet structure [36]. Figure 2d shows the HRTEM images of A-HC1100; the lattice fringes (0.379 nm) were significantly larger than the graphite layer spacing (0.335 nm), which may effectively promote the Na^+^ transport kinetics. The elemental mapping images show that the C and O elements were uniformly distributed in A-HC1100 (Figure 2e,f). The above results confirm that the oxygen-containing functional groups were successfully introduced in A-HC1100.

**Figure 3:**
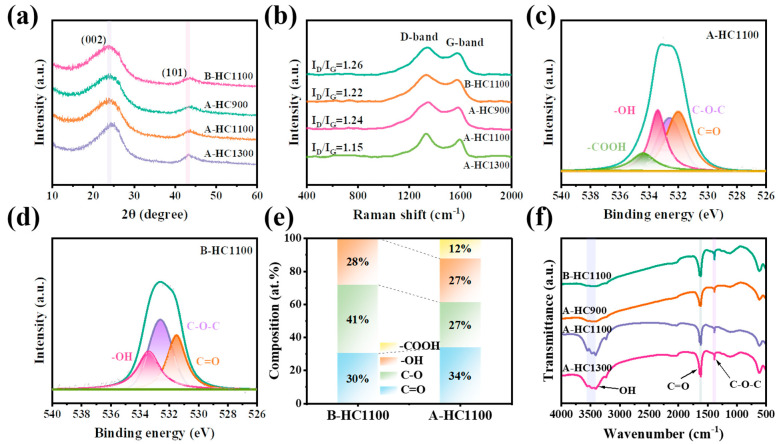
(**a**) XRD patterns of all samples; (**b**) Raman spectra of all samples. XPS O 1s spectra of (**c**) A-HC1100 and (**d**) B-HC1100. (**e**) Corresponding functional groups’ contributions of fitted O 1s spectra of A-HC1100 and B-HC1100. (**f**) FTIR spectra of A-HC1100, B-HC1100, A-HC900, and A-HC1300.

The crystalline structural features of the as-prepared samples were observed through XRD patterns. As shown in Figure 3a, all samples showed two major broad peaks at approximately 24° and 43°, corresponding to the (002) and (101) lattice planes of HC, respectively [37,38]. Interestingly, the peak (002) of A-HC1100 shifted to a higher angle than that of B-HC1100, indicating that the local structure of the material became more ordered. As is evident, Table 1 provides a summary of the value of d_002_ based on Bragg’s formula. As can be seen, the (002) peak shifted to a higher angle with the increasing carbonization temperature [39]. Figure 3b shows the Raman spectra of the samples, and every sample has two distinct and independent peaks, corresponding to the D band (~1340 cm^−1^) with defects and the G band (~1580 cm^−1^) with ordered graphite sp^2^ characteristics [40]. According to Table 1, the I_D_/I_G_ ratio indicates the degree of defect in the carbon material and the calculations [41,42]. Furthermore, below 1100 °C, the I_D_/I_G_ value of the oxidized sample increased, indicating that the local structure of the microcrystals was more disordered. While temperatures beyond 1100 °C caused the I_D_/I_G_ value to fall, this indicates that the material had a higher degree of graphitization and a more ordered structure, which may be related to the volatilization of functional groups at 1300 °C. These results are consistent with the results of other XRD and TEM analyses [43,44]. Therefore, it can be concluded that the degrees of crystallinity and graphitization of HC materials can be regulated by controlling the oxygen-containing functional groups and carbonization temperature. For further analysis of the porosity and specific surface area of A-HC1100 and B-HC1100, the nitrogen adsorption–desorption isotherm is displayed in Figure S5a. It can be clearly seen that the specific surface areas of all samples were low. This phenomenon may have been due to the anthracite’s original microspore—the A-HC1100 had a microspore structure, and the specific surface area was 3.88 m^2^ g^−1^. The specific surface area progressively dropped as the temperature of carbonization rose (Table 1), which was more likely to result in higher initial Coulombic efficiency (ICE) [45,46]. Figure S5b shows that the microspores were mainly concentrated in the range of 1.5–3 nm, and the pore size diminished gradually with the increase in temperature; this could be explained by the disintegration of OFGs and the increased stacking contact between graphene layers at elevated temperatures [47,48]. The pores can provide transport channels for electrolytes to promote ion transport dynamics and, thus, enhance the storage performance of Na^+^ [49]. For the purpose of analyzing the oxygen-containing functional groups, XPS was employed. The O 1s spectra (Figure 3c,d and Figure S6a,b) can be broken down into four peaks to represent the ether groups (C-O-C) at 532.6 eV; the carbonyl groups (C=O) at 531.6 eV; the hydroxy groups (-OH) at 533.4 eV; and the carboxyl (-COOH) groups at 534.4 eV, respectively [50,51,52]. Through a detailed analysis of the O 1s spectral functional group content, it is worth noting that the composition and content of the functional groups changed significantly before and after oxidation. It was found that -COOH emerged after oxidation, demonstrating that differences in oxygen content can affect the material’s structure. It is believed that the C=O functional groups are efficient and active sites for reversible Na-ion storage, and the C=O functional group of A-HC1100 is more abundant than that of B-HC1100 (Figure 3e). The Fourier transform infrared (FTIR) spectra of A-HC1100, B-HC1100, A-HC900, and A-HC1300 are shown in Figure 3f. The concurrence of the oxidized coal-based carbon materials exhibited similar FTIR spectra and different degrees of vibration peaks of the carboxylate hydroxyl group (-OH) at 3414 cm^−1^, the carbonyl group (C=O) at 1637 cm^−1^, and the ether (C-O-C) at 1383 cm^−1^ [53,54]. The above results demonstrate that the OFGs were effectively incorporated into HC and that they matched the XPS findings.

### 3.2. Electrochemical Performance

**Figure 4:**
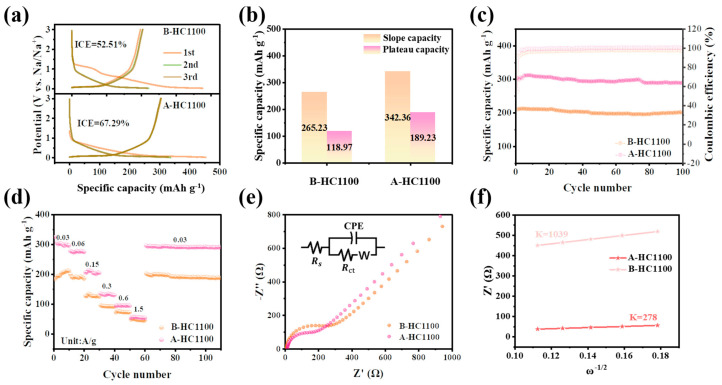
(**a**) GCD curves; (**b**) capacity contribution of the slope region and plateau region in the first-cycle discharge curve; (**c**) cycling performance; (**d**) rate performance; (**e**) EIS spectra of A-HC1100 and B-HC1100; and (**f**) slope of the fitted Z′ vs. ω^−1/2^ plots for A-HC1100 and B-HC1100.

The galvanostatic charge–discharge profiles of B-HC1100 and A-HC1100 at a current density of 0.015 A g^−1^ for the first, second, and third curves are shown in Figure 4a. As can be seen, A-HC1100 (67.29%) had a higher ICE than B-HC1100 (51.20%); furthermore, the first discharge and charge capacities of A-HC1100 were able to reach 454 and 305 mAh g^−1^, both of which were higher than those of B-HC1100 (376 and 197 mAh g^−1^). The Na^+^ representative intercalation/de-intercalation process in HCs can be divided into two phases, namely, the slope region above 0.1 V and the plateau region below 0.1 V. The capacity of the sloping region originated from sodium adsorption on the defect sites, functional groups, and other doped heteroatoms [55,56]. Figure 4b and Table S2 show the capacity contribution rate calculated from the first charge–discharge curve at 0.03 A g^−1^. As can be seen, the capacity contribution of the two electrodes primarily came from the high-pressure slope region; in the first discharge, A-HC1100 had a higher sloping region capacity and plateau capacity, reaching 342 mAh g^−1^ and 189 mAh g^−1^. In contrast, B-HC1100 had a lower sloping capacity and plateau capacity, reaching 265 and 118 mAh g^−1^, respectively. This increase in capacity may be attributed to the increase in C=O, which may have provided an effective active site for reversible sodium storage. Figure 4c and Figure S7a show the cycling performance of A-HC1100 and B-HC1100 at 0.03 A g^−1^; A-HC1100 showed a relatively higher reversible capacity (304.20 mAh g^−1^) than that of B-HC1100, indicating that C=O can provide an effective active site for Na^+^ storage. Figure 4d shows the rate performance of A-HC1100 and B-HC1100. When the current density values were 0.03, 0.06, 0.15, 0.3, 0.6, and 1.5 A g^−1^, the corresponding charging capacities of A-HC1100 were 305, 277, 210, 135.16, 96, and 56 mAh g^−1^, all of which were higher than those of B-HC1100. Furthermore, when the low-current density returned, the specific charging capacity was 295.49 mAh g^−1^ and the capacity retention rate was 96.74%, while the capacity retention rate of B-HC1100 was only 92.71%, indicating the high reversibility and structural stability of A-HC1100 during sodium-ion insertion and extraction.

To further explore the electrode kinetics of hard carbons, the electrochemical impedance spectrum (EIS) was employed as shown in Figure 4e and Figure S8. The Nyquist diagram is fitted from the equivalent circuit model (R(C(RW)) and consists of four parts, corresponding to the electrolyte-related ohm resistance (R_s_), charge transfer resistance (R_ct_), CPE, and Warburg impedance (W) [3,54]. Apparently, the A-HC1100 anode has a lower R_ct_ (140 Ω) than that of B-HC1100 (221 Ω). To strengthen the results, the Na-ion diffusion coefficient (D_Na+_) can be calculated from the low-frequency plots by the following Equations (1) and (2).

(1) $$D_{{\mathrm{Na}}^{+}}=R^{2}T^{2}/(2A^{2}F^{4}n^{4}C^{2}\sigma^{2}),$$

(2) $$Z^{\prime }=R_{s}+R_{\mathrm{ct}}+R_{f}+\sigma \omega^{-1/2}$$

where D_Na+_, R, and T are the diffusion coefficients of Na^+^, the gas constant, and the absolute temperature, respectively. The surface area of the anode, the Faraday constant, the number of electrons per molecule throughout the reaction, the concentration of Na^+^, and the simulation of the Warburg factor are represented by the variables A, F, n, C, and σ, respectively [57,58]. The Warburg factor (σ) value of the B-HC1100 is about four times higher than that of A-HC1100 obtained from the slope of the Z′ vs. ω^−1/2^ plots (Figure 3f) according to Equation (2), indicating that A-HC1100 has a much higher D_Na+_ (2.68 × 10^−15^ cm^2^ s^−1^) than that of B-HC1100 (7.17 × 10^−16^ cm^2^ s^−1^) from Equation (1). The lower R_ct_ and higher D_Na+_ of A-HC1100 show that the oxygen-containing functional groups in A-HC1100 can improve the charge transfer and D_Na+_ diffusion.

Figure S9a,b shows the CV curves of B-HC1100 and A-HC1100 at a scan rate of 0.1 mV s^−1^. Around 1.05 V and 0.30 V in the initial cycle, an irreversible reduction peak emerged. This can be explained by the side reactions that occurred during the first Na^+^ intercalation and the decomposition of the electrolytes, as well as the appearance of a solid electrolyte interface (SEI) film on the surface of the electrode [59,60]. The second irreversible reduction peak occurred around 0.50 V, which was probably due to the irreversible reaction (C=O + Na^+^+ e^−^→ C-O-Na). A set of redox peaks at approximately 0.01 V and 0.25 V corresponded to Na^+^ insertion and de-intercalation between interlamellar graphite. Compared with B-HC1100, the subsequent curve for A-HC1100 had a higher overlap degree, indicating that the electrochemical reaction in the A-HC1100 electrode was reversible and the structure was stable.

**Figure 5:**
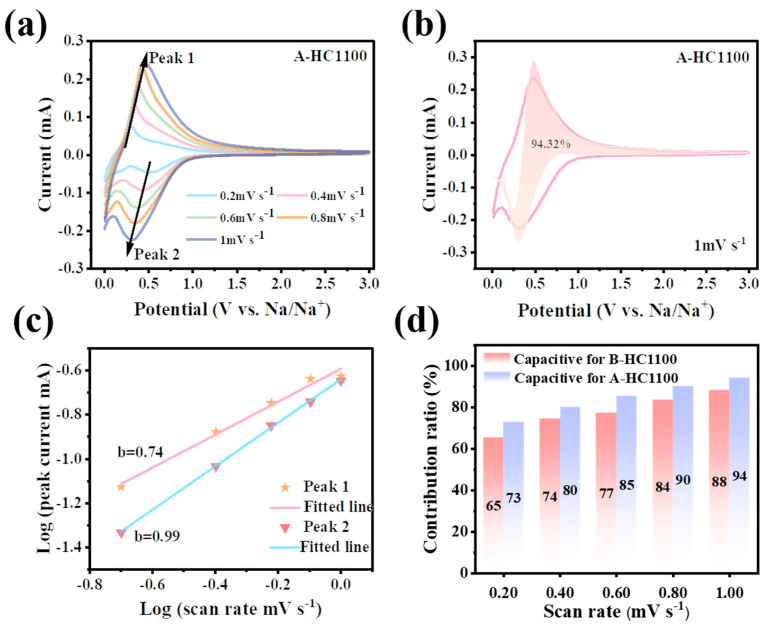
(**a**) CV curves at various scan rates from 0.2 to 1.0 mV s^−1^ for A-HC1100; (**b**) capacitive contribution at a 1.0 mV s^−1^ scan rate; (**c**) the linear relationship between log i and log v; and (**d**) the contribution ratios of the capacitive and non-capacitive charge versus the scan rate.

Figure 5a and Figure S10a display the CV curves of A-HC1100 and B-HC1100 at various scan rates from 0.2 to 1.0 mV s^−1^, with the curves showing similar shapes and peak profiles. The two primary processes thought to be responsible for controlling the electrodes’ capacity contribution are the diffusion-controlled insertion process and the surface-controlled pseudocapacitive behavior. Equation (3) illustrates the correlation between the peak current and the scan rate, which can be used to predict the sodium storage behavior: i is the peak current, v is the scan rate, and a and b are the adjustable parameters. The sodium-ion storage behavior can be reflected by the b values in different potential regions [61]. When the b-value is in the range of 0.5–1, this proves that the above two contribution behaviors exist simultaneously [62,63]. Figure 5c and Figure S10c exhibit the linear relationship between log (i) and scanning log (v) according to Equation (3); the b value was close to 1 in all cases, confirming that capacitance is an essential part of the storage mechanism [64]. Equation (4) can be applied to further identify both capacitive ($k_{1}v$) and diffusion-controlled current responses ($k_{2}v^{1/2}$) [65].

(3) $$i=av^{b},$$

(4) $$i\left(V\right)=k_{1}v+k_{2}v^{1/2}$$

According to the quantitative analyses, the capacitive contribution of A-HC1100 to the overall capacity was determined to be 94.32% (Figure 5b) at a scan rate of 1 mV s^−1^, which is higher than that of B-HC1100 (88.23%), as shown in Figure S10b. Furthermore, as can be seen from Figure 5d, the A-HC1100 contribution ratios of capacitance were 72.72%, 80.11%, 85.49%, 90.06%, and 94.32% at scan rates from 0.2 mV s^−1^ to 1.0 mV s^−1^, which are all higher than that of B-HC1100 (65.24%, 74.32%, 77.28%, 83.50%, and 88.23%) shown in Figure S10d, indicating the excellent rate capability of A-HC1100. The expanding D_Na+_ and mesopore structures of A-HC1100 could be the cause of the slightly greater ratio of the capacitive contribution [66,67,68].

## 4. Conclusions

In summary, hard carbon with abundant oxygen-containing functional groups was achieved by combining pre-oxidation anthracite and controlling the carbonization temperature. The oxygen-containing functional groups provided apparent defects, improved the capacity of the tilt zone, and produced more adsorption sites. As a result, the optimal sample presented a high initial reversible sodium storage capacity of 304 mAh g^−1^ at 0.03 A g^−1^, with an ICE of 67.29% and an excellent capacitance retention of 95.17% after 100 cycles. Moreover, this work provides a green and convenient method for designing SIB anode materials with high performance and low cost, which will be favorable for large-scale energy storage from the perspective of adjusting the surface oxygen content in the precursor.

## Figures and Tables

**Table 1:** Structural parameters estimated for the samples from the XRD, Raman, and nitrogen adsorption–desorption results.

Sample Name	B-HC1100	A-HC900	A-HC1100	A-HC1300
$d_{002}$(Å)	3.75	3.70	3.72	3.67
$I_{D}$/$I_{G}$	1.26	1.22	1.24	1.15
$S_{\mathrm{BET}}$($m^{2}$ $g^{-1}$)	1.14	12.44	3.88	1.63

## Data Availability

Data are contained within the article and Supplementary Materials.

## Supplementary Materials

The following supporting information can be downloaded at: https://www.mdpi.com/article/10.3390/nano13233002/s1. Figure S1: (**a**) The XPS full spectrum and (**b**) XRD pattern of A-HC1100. Figure S2: FESEM images of (**a**) B-HC1100, (**b**) A-HC900, and (**c**) A-HC1300. Figure S3: TEM images of (**a**) B-HC1100, (**b**) A-HC900, and (**c**) A-HC1300. Figure S4: TEM images of (**a**) B-HC1100, (**b**) A-HC900, and (**c**) A-HC1300. Figure S5: (**a**) N_2_ adsorption–desorption isotherms and (**b**) pore size distribution. Figure S6: Fitted XPS O 1s spectra of (**a**) A-HC900, (**b**) A-HC1300, and (**c**) corresponding functional group contributions of the fitted O 1s spectra. Figure S7: (**a**) Cycling performance and (**b**) rate performance of A-HC900 and A-HC1300. Figure S8: EIS spectra of A-HC900 and A-HC1300. Figure S9: CV curves recorded at a scan rate of 0.1 mV s^−1^ of (**a**) B-HC1100 and (**b**) A-HC1100. Figure S10: (**a**) CV curves at various scan rates from 0.2 to 1.0 mV s^−1^ for B-HC1100; (**b**) contribution of capacitive at a 1.0 mV s^−1^ scan rate; (**c**) the linear relationship between log i and log v; (**d**) contribution ratios of the capacitive and non-capacitive charge versus the scan rate. Table S1: EDS element analysis of pristine anthracite and pretreated anthracite. Table S2: The first cycle electrochemical performance of A-HCs electrodes. Table S3: A brief summary of the preparation technology electrochemical measurement condition and battery performance of anthracite. See Refs. [20,29,69,70,71,72,73].
