# Supplementary material for: Constructing Abundant Oxygen-Containing Functional Groups in Hard Carbon Derived from Anthracite for High-Performance Sodium-Ion Batteries
Source: Nanomaterials (Basel). 2023 Nov 22;13(23):3002. doi: 10.3390/nano13233002 (PMC10708234; doi:10.3390/nano13233002)
Supplement: Supplementary file 1 [file nanomaterials-13-03002-s001.zip › nanomaterials-2710368-supplementary.pdf]

Supporting Information

The anthracite used in our work was pretreated. The impurities (such as silica and alumina) in anthracite were almost removed according to the EDS, XPS and XRD analysis [1]. The EDS element analysis of pristine anthracite and pretreated anthracite were shown in Table. s1, as can be seen, the pretreated anthracite has less silica and alumina impurity content. The XPS full spectrum (Fig. s1a) shows that the main elements in the A-HC1100 (synthesis by using pretreated anthracite as precursor) are C and O. The XRD pattern (Fig. s1b) of A-HC1100 show two major peaks at approximately 24°and 43°, corresponding to the (002) and (101) lattice planes of HC without any impurity phases. All of the above proves that the impurity has been removed.

Table S1. EDS element analysis of pristine anthracite and pretreated anthracite.

Sample	C (wt %)	Au (wt %)	O (wt %)	Al (wt %)	Si (wt %)	S (wt %)	Fe (wt %)
Pristine anthracite	83.78	11.56	4.05	0.04	0.06	0.46	0.05
Pretreated anthracite	86.26	9.31	4.16	0.02	0.02	0.23	0

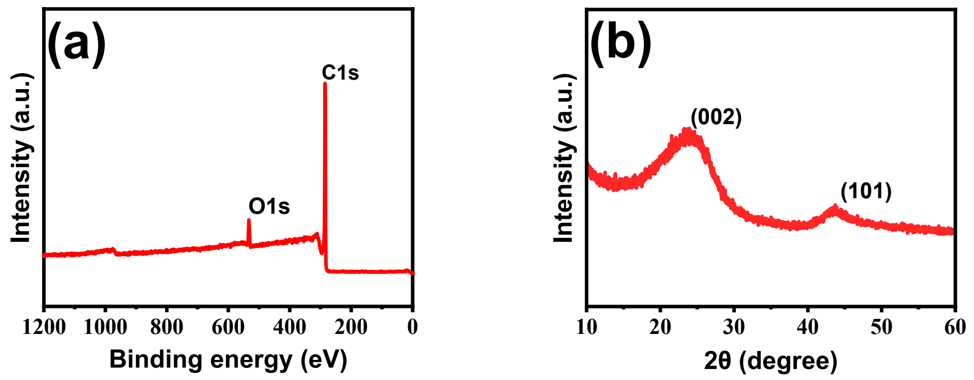

Figure S1 (**a**) The XPS full spectrum and (**b**) XRD pattern of A-HC1100.

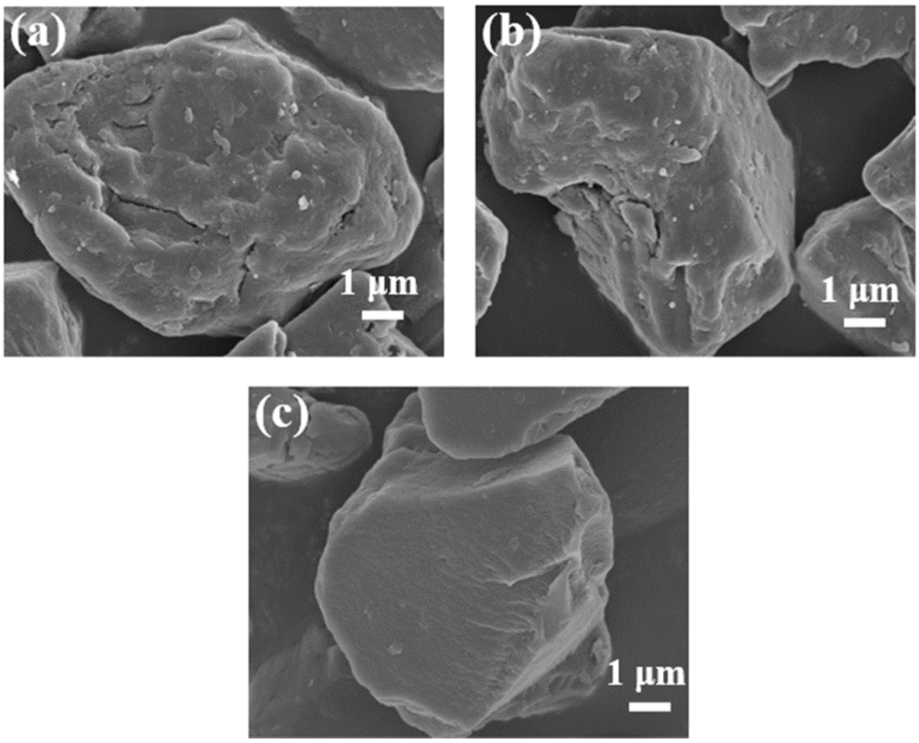

Figure S2. FESEM images of (**a**) B-HC1100, (**b**) A-HC900, and (**c**) A-HC1300.

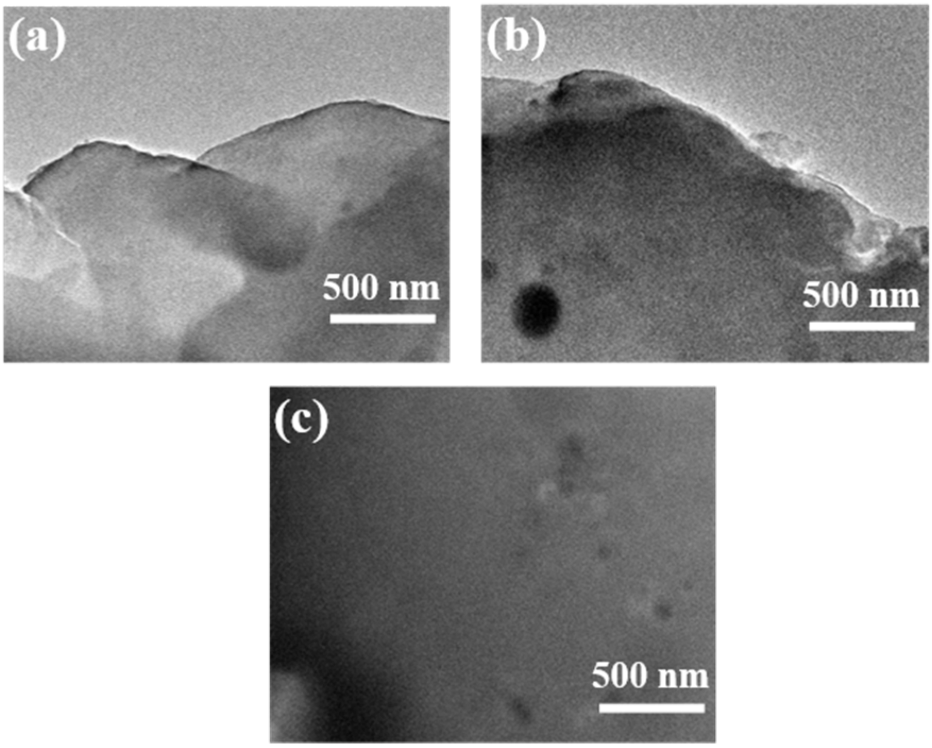

Figure S3. TEM images of (**a**) B-HC1100, (**b**) A-HC900, and (**c**) A-HC1300.

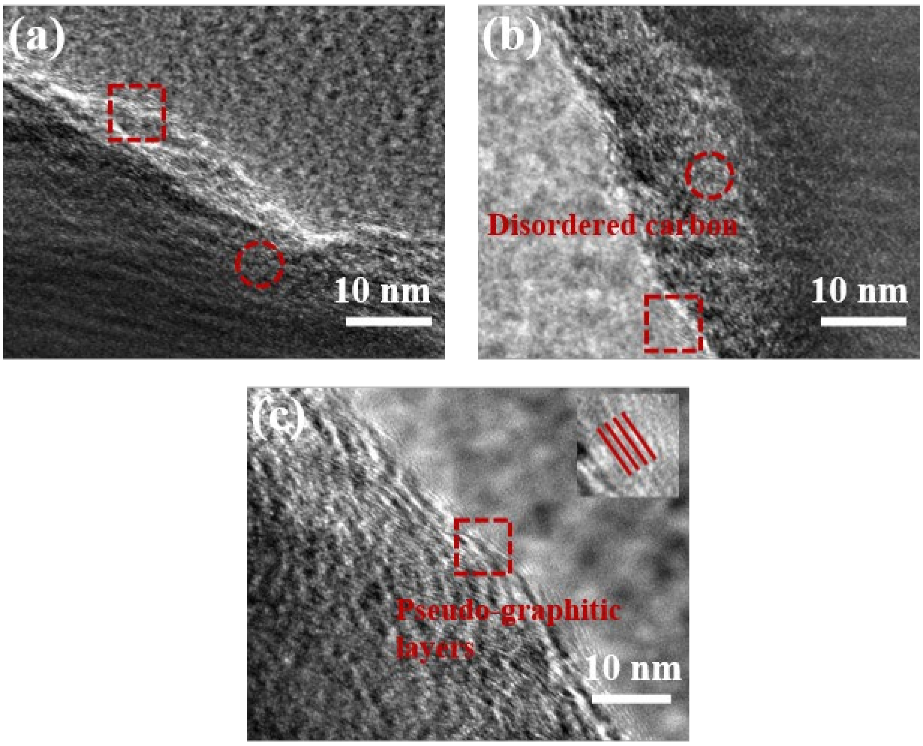

Figure S4. TEM images of (**a**) B-HC1100, (**b**) A-HC900, and (**c**) A-HC1300.

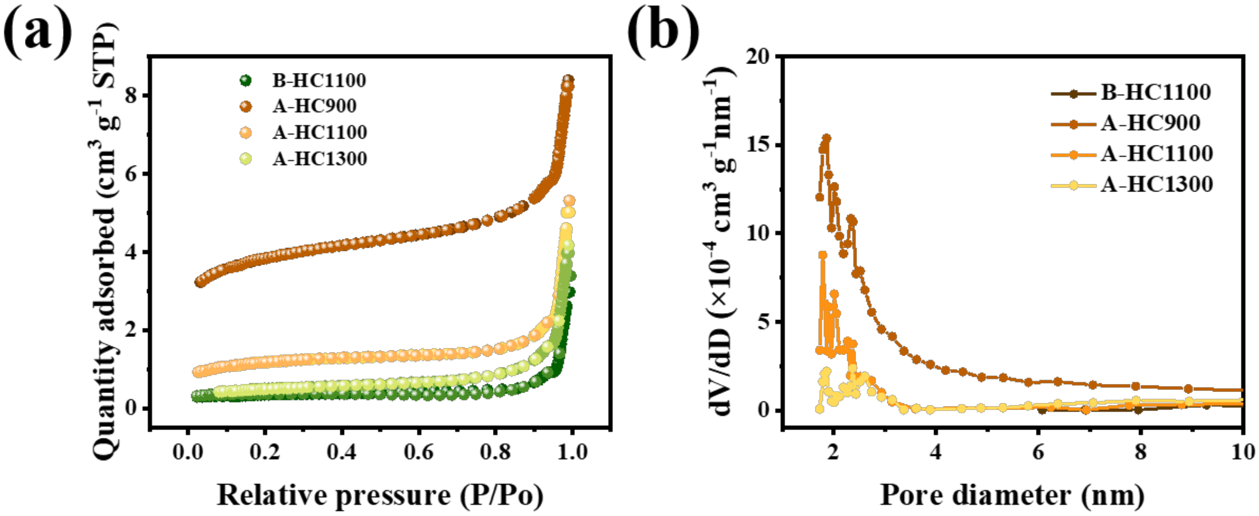

Figure S5. (**a**) N_2 adsorption–desorption isotherms, and (**b**) pore size distribution.

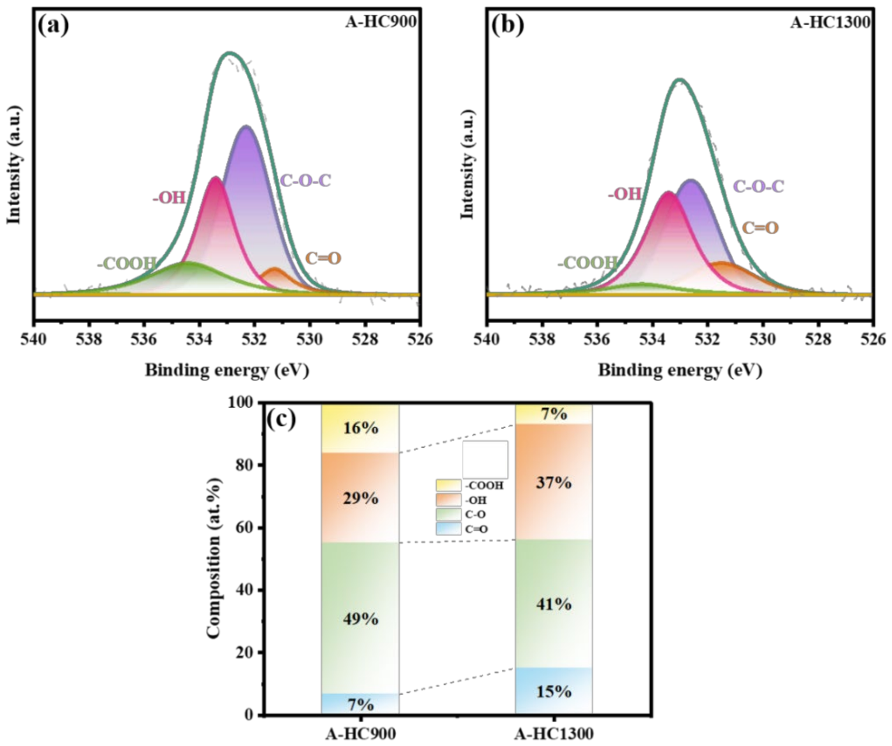

Figure S6. Fitted XPS O 1s spectra of (**a**) A-HC900, (**b**) A-HC1300, and (**c**) corresponding functional group contributions of the fitted O 1s spectra.

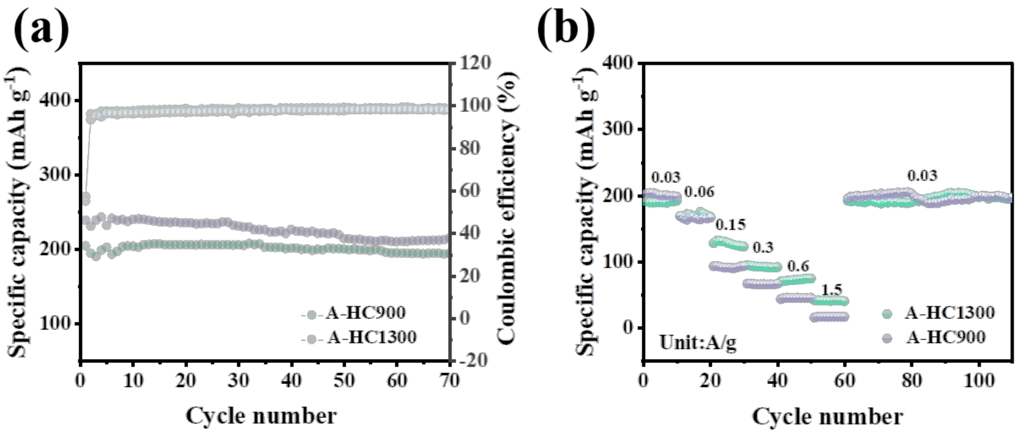

Figure S7. (**a**) Cycling performance and (**b**) rate performance of A-HC900 and A-HC1300.

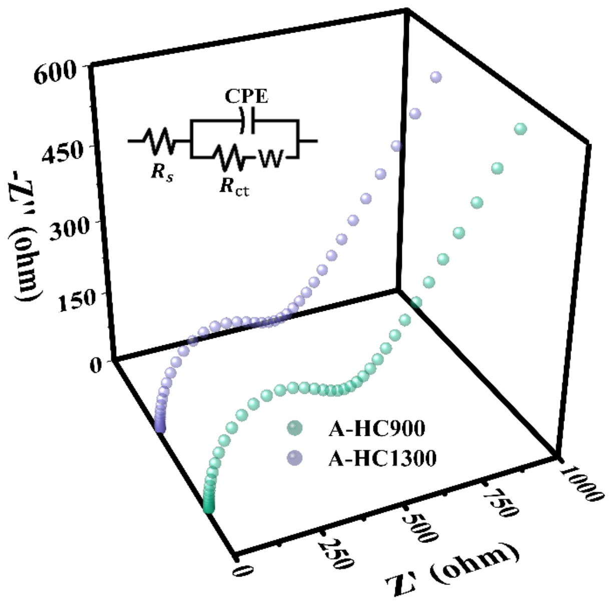

Figure S8. EIS spectra of A-HC900 and A-HC1300.

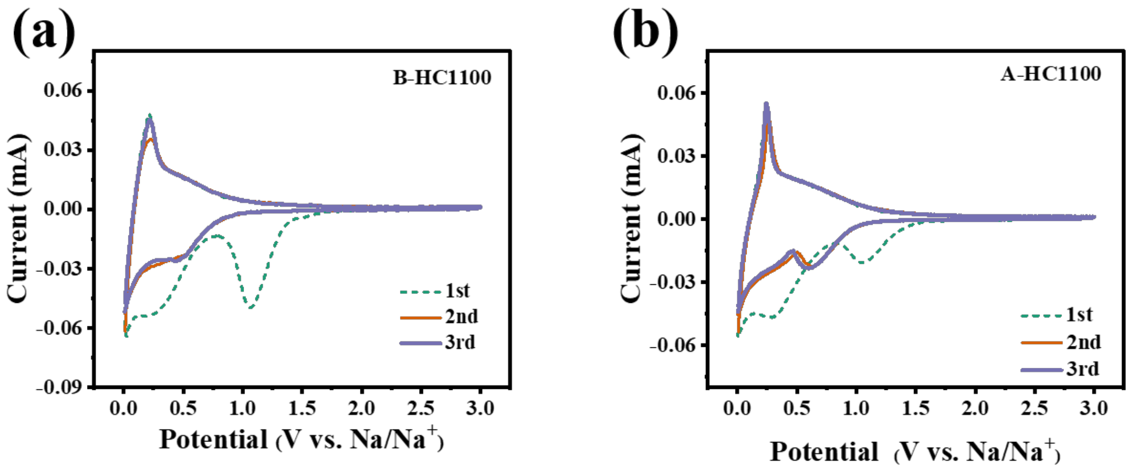

Figure S9. CV curves recorded at a scan rate of 0.1 mV s^{-1} of (**a**) B-HC1100 and (**b**) A-HC1100.

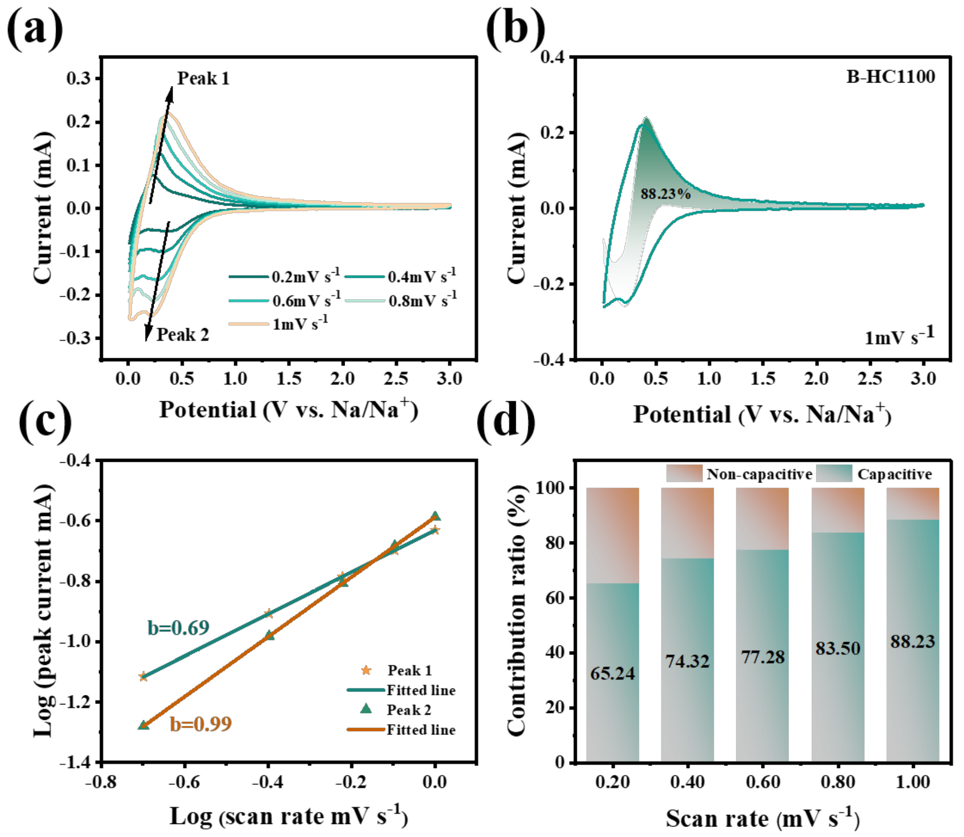

Figure S10. (**a**) CV curves at various scan rates from 0.2 to 1.0 mV s^{-1} for B-HC1100; (**b**) contribution of capacitive at a 1.0 mV s^{-1} scan rate; (**c**) the linear relationship between log i and log v; (**d**) contribution ratios of the capacitive and non-capacitive charge versus the scan rate.

Table S2. The first cycle electrochemical performance of A-HCs electrodes.

**Sample**	**Discharge (mAh g^{-1})**	**Charge (mAh g^{-1})**	**ICE (%)**	**Sloping (mAh g^{-1})**	**Plateau (mAh g^{-1})**
B-HC1100	384	210	54	265	118
A-HC900	344	204	59	254	89
A-HC1100	531	304	57	342	189
A-HC1300	433	238	55	277	155

Table S3. A brief summary of the preparation technology electrochemical measurement condition and battery performance of anthracite.

Precursor	Preparation technology	Current (A g^{-1})	Capacity (mAh g–1) after (n) cycles	Ref.
Anthracite	Acid pickling	0.05	267.7(10)	[2]
Anthracite	Direct pyrolysis	0.3	170(200)	[3]
Anthracite	Acid etching and oxygen functionalization	0.03	175(50)	[4]
Anthracite-derived carbon	Acid pickling	1	81.3(500)	[5]
Anthracite	Hydrogenation and pyrolysis	0.02	307(10)	[6]
Anthracite	Acid pickling	0.05	138(80)	[7]
Pristine anthracite coal	Heat treatment	0.037	160(100)	[8]
Anthracite	Pre-oxidation	0.03	304.20(100)	This work

Supplementary references

1. Wang, B.; Xia, J.; Dong, X.; Wu, X.; Jin, L.; and Li, W. Highly purified carbon derived from deashed anthracite for sodium-ion storage with enhanced capacity and rate performance. *Energy Fuels* **2020**, 34, 16831-16837.
2. Quan, L.; Guo, Y.; Wen, H. Investigation of pyrolysed anthracite as an anode material for sodium ion batteries. *New J. Chem.* **2022**, 46(28), 13575-81.
3. Li, Y.; Hu, Y.; Qi, X.; Rong, X.; Li, H.; Huang, X.; Chen, L. Advanced sodium-ion batteries using superior low cost pyrolyzed anthracite anode: towards practical applications. *Energy Stor Mater.* **2016**, 5, 191-7.
4. Zhao, H.; Zhao, D.; Ye, J.; Wang, P.; Chai, M.; and Li, Z. Directional oxygen functionalization by defect in different metamorphic-grade coal-derived carbon materials for sodium storage. *Energy Environ. Mater.* **2021**, 5(1), 313-20.
5. LI, F.; Tao, H.; Liu, X.; Yang. X.; Pyrolyzed hydrogenated anthracite as anode materials for sodium-ion batteries. *Chin. Ceramic. Soc.* **2022**, 50(7): 1890-1898.
6. Zhuang, Z.; Cui, Y.; Zhu, H.; Shi, Y.; Zhuang, Q. Coal-based amorphous carbon as economical anode material for sodium-ion battery. *J. Electro chem. Soc.* **2018**, 165(10): A2225.
7. Le, M.; Tran, T.; Huynh, T.; Nguyen, V.; Vo, D.; Tran, V.; Le, M. Development of vang danh anthracite as a costeffective anode for sodium-ion batteries through a heat-treatment process. *RSC Adv.* **2022**, 12(46): 29900-7.
